# Integrated diagnosis based on transcriptome analysis in suspected pediatric sarcomas

**DOI:** 10.1038/s41525-021-00210-y

**Published:** 2021-06-15

**Authors:** Daisuke Ichikawa, Kyoko Yamashita, Yusuke Okuno, Hideki Muramatsu, Norihiro Murakami, Kyogo Suzuki, Daiei Kojima, Shinsuke Kataoka, Motoharu Hamada, Rieko Taniguchi, Eri Nishikawa, Nozomu Kawashima, Atsushi Narita, Nobuhiro Nishio, Asahito Hama, Kenji Kasai, Seiji Mizuno, Yoshie Shimoyama, Masato Nakaguro, Hajime Okita, Seiji Kojima, Atsuko Nakazawa, Yoshiyuki Takahashi

**Affiliations:** 1 Department of Pediatrics, Nagoya University Graduate School of Medicine, Nagoya, Japan; 2 Department of Pathology and Biological Responses, Nagoya University Graduate School of Medicine, Nagoya, Japan; 3 Department of Pathology, The Cancer Institute Hospital, Japanese Foundation for Cancer Research, Tokyo, Japan; 4 Medical Genomics Center, Nagoya University Hospital, Nagoya, Japan; 5 Department of Advanced Medicine, Center for Advanced Medicine and Clinical Research, Nagoya University Hospital, Nagoya, Japan; 6 Department of Pathology, Aichi Medical University School of Medicine, Nagakute, Japan; 7 Department of Pediatrics, Central Hospital, Aichi Developmental Disability Center, Kasugai, Japan; 8 Department of Pathology and Laboratory Medicine, Nagoya University Hospital, Nagoya, Japan; 9 Department of Pathology, National Center for Child Health and Development, Tokyo, Japan; 10 Division of Diagnostic Pathology, Keio University School of Medicine, Tokyo, Japan; 11 Department of Clinical Research, Saitama Children’s Medical Center, Saitama, Japan

**Keywords:** Paediatric cancer, RNA sequencing

## Abstract

Pediatric solid tumors are a heterogeneous group of neoplasms with over 100 subtypes. Clinical and histopathological diagnosis remains challenging due to the overlapping morphological and immunohistochemical findings and the presence of atypical cases. To evaluate the potential utility of including RNA-sequencing (RNA-seq) in the diagnostic process, we performed RNA-seq in 47 patients with suspected pediatric sarcomas. Histopathologists specialized in pediatric cancer re-evaluated pathological specimens to reach a consensus diagnosis; 42 patients were diagnosed with known subtypes of solid tumors whereas 5 patients were diagnosed with undifferentiated sarcoma. RNA-seq analysis confirmed and refined consensus diagnoses and further identified diagnostic genetic variants in four of the five patients with undifferentiated sarcoma. Genetic lesions were detected in 23 patients, including the novel *SMARCA4*-*THOP1* fusion gene and 22 conventional or recently reported genetic events. Unsupervised clustering analysis of the RNA-seq data identified a distinct cluster defined by the overexpression of rhabdomyosarcoma-associated genes including *MYOG* and *CHRNG*. These findings suggest that RNA-seq-based genetic analysis may aid in the diagnosis of suspected pediatric sarcomas, which would be useful for the development of stratified treatment strategies.

## Introduction

Pediatric solid tumors are a diverse group of neoplasms^1^, and accurate diagnosis of tumor subtype is necessary for appropriate patient management^2^. Disease-specific tumor markers, such as urine vanillylmandelic acid and homovanillic acid in neuroblastoma, have diagnostic value^3^; however, most tumors lack specific tumor markers and the diagnosis is strongly dependent on histopathological evaluation. Most tumors exhibit undifferentiated small round-cell morphology, requiring ancillary studies for diagnosis. Accordingly, several immunomarkers, including NK2 homeobox 2 (NKX2.2) and cluster of differentiation 99 (CD99) for Ewing sarcoma (ES)^4,5^, desmin and myogenin for rhabdomyosarcoma^5^, and SWI/SNF-related matrix-associated actin-dependent regulator of chromatin (SMARC) subfamily B member 1 (SMARCB1) for malignant rhabdoid tumor, can be used to distinguish tumor subtypes^6^. In addition, molecular studies, including reverse transcription (RT)-polymerase chain reaction (PCR) and fluorescence in situ hybridization (FISH), can be used to assist the diagnostic process. However, the diagnosis remains challenging for many pediatric solid tumors due to overlapping morphological and/or immunohistochemical features; presence of unusual clinical, morphological, and immunohistochemical features; and the availability of limited biopsy specimens^7^.

The detection of disease-specific genetic alterations, especially the identification of specific fusion genes such as *EWSR1*-*FLI1* in ES^8^ and *PAX3/7*-*FOXO1* in alveolar rhabdomyosarcoma^9^, can improve the diagnosis of pediatric sarcomas. In addition, recent advances in molecular biological techniques have identified several subtype-defining somatic genetic alterations, including *BCOR*-*CCNB3*^10^ and *CIC*-*DUX4*^11^ in undifferentiated small round-cell sarcoma, *ZNF532*-*NUTM1* in NUT carcinoma^12^, internal tandem duplication of *BCOR* in clear cell sarcoma of the kidney^13^, and *MYOD1* p.Leu122Arg (p.L122R) in spindle cell/sclerosing rhabdomyosarcoma^14^.

In the present pilot study, we performed transcriptome analysis using RNA-sequencing (RNA-seq) to assess its clinical utility in the differential diagnosis of suspected pediatric sarcomas.

## Results

### Detection of diagnostic genetic lesions

We performed transcriptome analysis of 47 patients with 18 types of pediatric solid tumors with suspected sarcoma diagnosis (Table 1) and detected diagnostic genetic events in 23 of the 47 (49%) patients. Of these, 22 genetic events were conventional or recently reported alterations, including *EWSR1*-*FLI1*^8^, *PAX3*-*FOXO1*^9^, *SS18*-*SSX2*^15^, and *MYOD1* p.L122R^14^ in 6, 5, 2, and 2 patients, respectively, and *EWSR1*-*ETV1*^15^, *EWSR1*-*ATF1*^15^, *ZNF532*-*NUTM1*^12^, *BCOR*-*CCNB3*^16^, *TPM4*-*ALK*^15^, *PTCH1*-*GLI1*^17^, and *SRF-NCOA1*^18^ in one patient each. Additionally, we identified a novel fusion gene, *SMARCA4-THOP1*, in one patient with undifferentiated sarcoma (unique patient number [UPN] 66), which resulted in the truncation and subsequent loss-of-function of the SMARCA4 protein.

**Table 1:** Patient characteristics.

	Total cohort (*N* = 47)	Patients with genetic alterations	Detected alterations (cases)
Median age at initial diagnosis, months (range)	70 (1–215)		
Sex (male/female)	23/24		
Consensus histopathological diagnosis			
RMS, embryonal	7	0	
RMS, alveolar	6	5	*PAX3-FOXO1* (5)
RMS, spindle cell/sclerosing	3	3	*SRF-NCOA1* (1), *MYOD1* p.L122R (2)
RMS, NOS	3	0	
Ewing sarcoma	8	7	*EWSR1-FLI1* (6), *EWSR1-ETV1* (1)
Undifferentiated sarcoma	5	4	*EWSR1-ATF1 (1), PTCH1-GLI1 (1) SS18-SSX2 (1), SMARCA4-THOP1 (1)*
Malignant rhabdoid tumor	3	0	
Myxopapillary ependimoma	2	0	
Clear cell sarcoma of the kidney	1	1	*BCOR-CCNB3* (1)
Fetal rhabdomyomatous nephroblastoma	1	0	
Inflammatory myofibroblastic tumor	1	1	*TPM4-ALK* (1)
Langerhans cell histiocytosis	1	0	
Liposarcoma	1	0	
MPNST	1	0	
Neuroblastoma	1	0	
NUT carcinoma	1	1	*ZNF532-NUTM1* (1)
Subcutaneous panniculitis-like T-cell lymphoma	1	0	
Synovial sarcoma	1	1	*SS18-SSX2* (1)

*RMS,* rhabdomyosarcoma; *NOS,* not otherwise specified; *MPNST,* malignant peripheral nerve sheath tumor.

### Comparison of diagnoses without and with RNA-seq

All patients had institutional diagnoses based on the clinical information and pathological examination at the time of disease onset (1990–2018). Without the information of RNA-seq analyses, histopathologists specialized in pediatric solid tumors (A.N., M.N., and K.Y.) re-evaluated the histopathological diagnoses using a diagnostic flow-chart reflecting the current diagnostic criteria (consensus diagnosis without RNA-seq information in Supplementary Fig. 1 and Supplementary Table 1). The two diagnoses were discrepant in 16 of the 47 (34%) patients. The diagnosis was changed in four patients due to a change in the histopathological classification over time (UPNs 7, 15, 37, and 60) and modified in the remaining twelve patients due to improvements in pathological diagnostic techniques, including immunostaining and FISH (UPNs 4, 8, 21, 25, 26, 36, 51, 53, 57, 61, 64, and 66). The details of these patients are summarized in Supplementary Table 4.

**Fig. 1:**
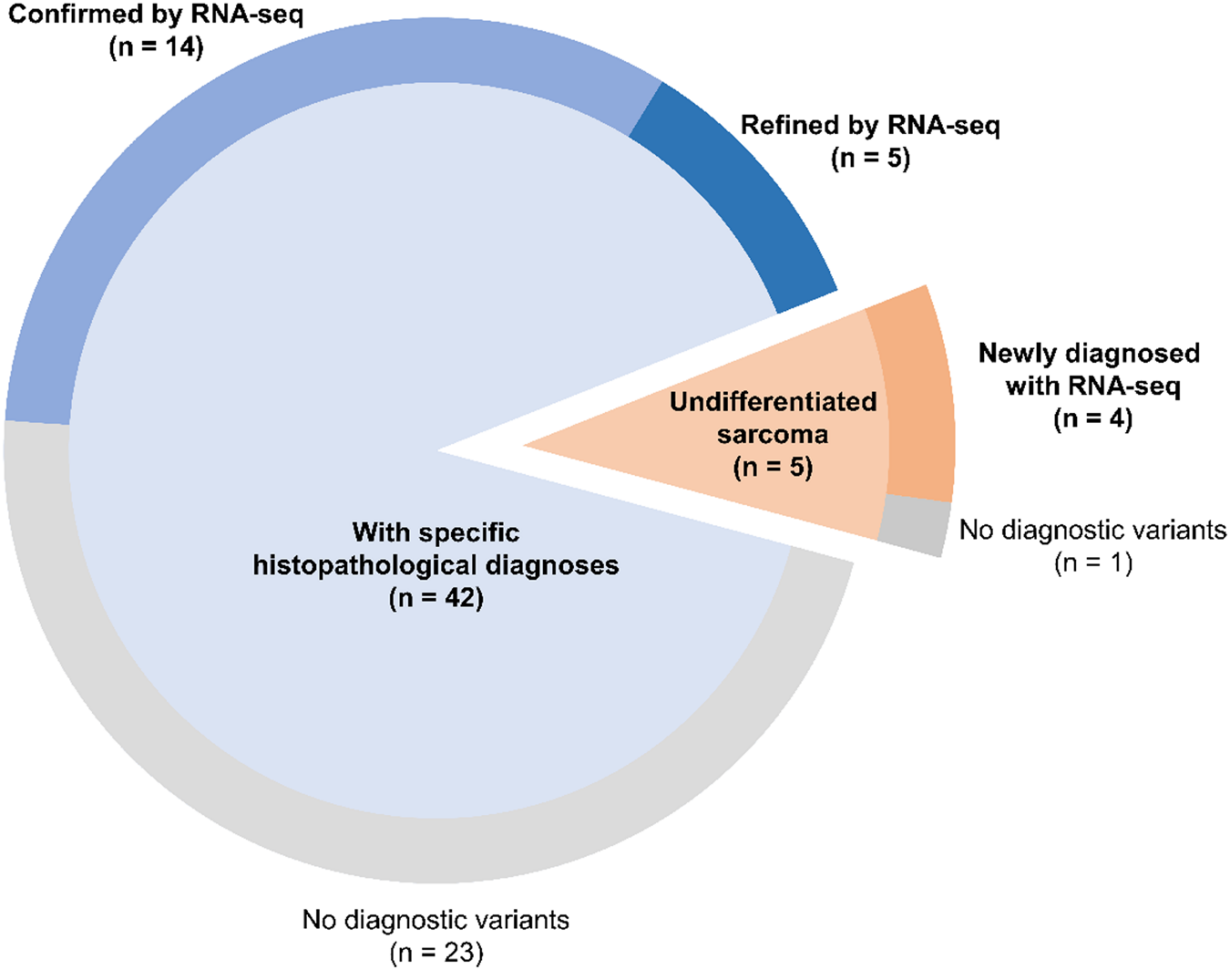
Clinical value of genetic analysis by RNA-seq. During the consensus histopathological review of incorporating fluorescence in situ hybridization analysis, 42 of 47 (89%) patients were diagnosed with known specific solid tumor subtypes whereas the remaining 5 (11%) patients were diagnosed with undifferentiated sarcoma. RNA-seq analysis confirmed and refined histopathological diagnosis in 14 and 5 patients, respectively, and identified novel diagnostic genetic variants in four of the five patients with undifferentiated sarcoma.

Next, we reached diagnoses by integrating the genetic information identified by RNA-seq with the consensus diagnosis (consensus diagnosis with RNA-seq information). During the consensus histopathological review, 5 of the 47 (11%) patients were diagnosed with undifferentiated sarcoma. The histopathological features of these five patients did not match any known pathological features of specific solid tumor subtypes. Genetic alterations supporting the genetic diagnosis were identified in four of the five patients (Fig. 1), including *EWSR1-ATF1* in clear cell sarcoma*, PTCH1-GLI1* in *GLI1*-rearranged tumor*, SS18-SSX2* in synovial sarcoma, and *SMARCA4-THOP1* in SMARCA4-deficient undifferentiated sarcoma. Moreover, the RNA-seq results refined the consensus diagnosis in 5 (11%) additional patients (Fig. 1), including *SRF-NCOA1* in spindle cell rhabdomyosarcoma, *MYOD1* p.L122R in sclerosing rhabdomyosarcoma, *TPM4-ALK* in inflammatory myofibroblastic tumor, and *BCOR-CCNB3* in clear cell sarcoma of the kidney. Overall, RNA-seq improved the consensus diagnosis in 9 (19%) patients (Fig. 1). The RNA-seq analysis also confirmed the consensus diagnosis in 14 (30%) additional patients. Candidate genetic alterations were validated by Sanger sequencing of the RT-PCR products (Supplementary Fig. 2)

### *GLI1*-rearranged tumor with *PTCH1-GLI1* fusion: UPN 36

**Fig. 2:**
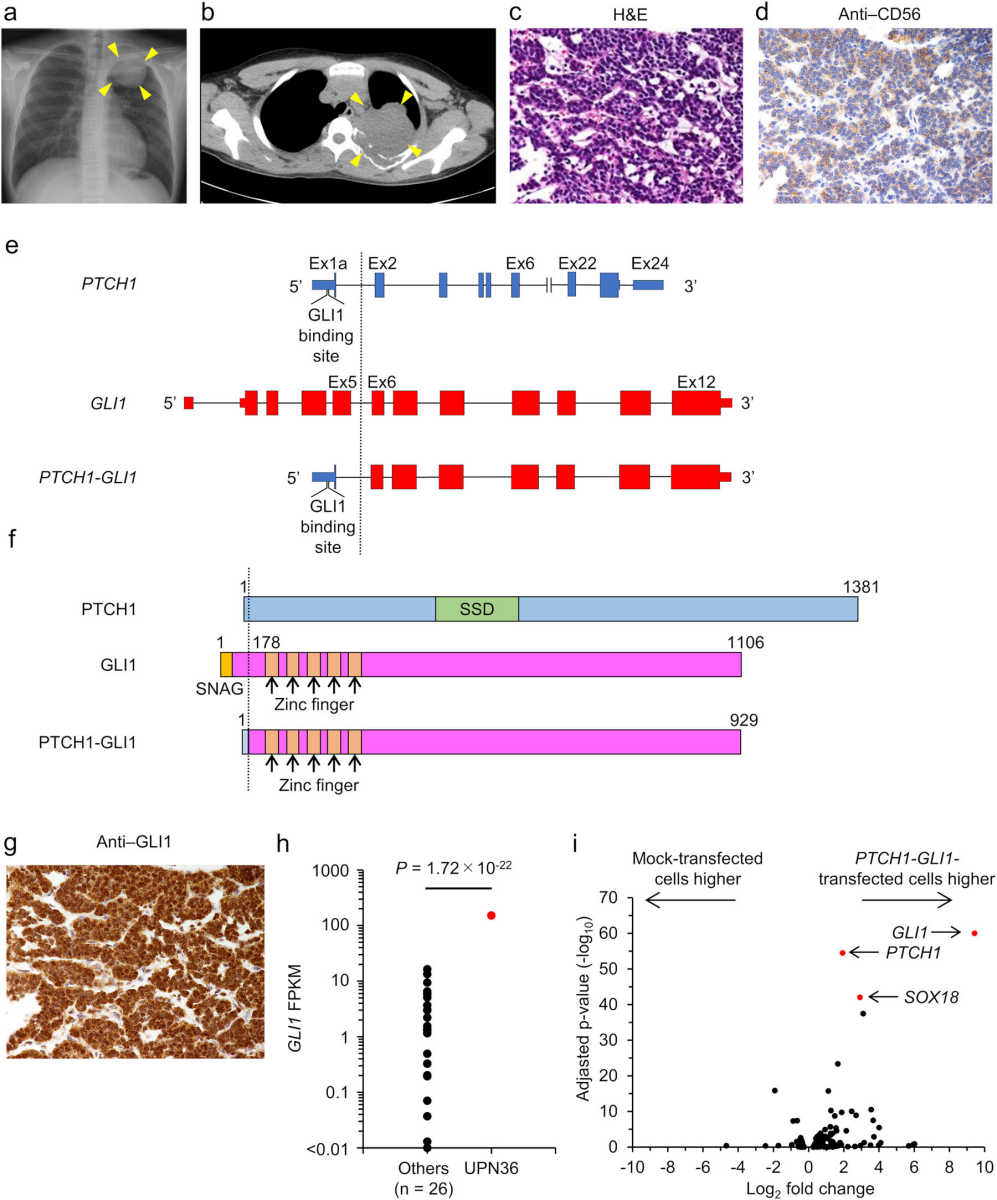
*GLI1*-rearranged tumor with *PTCH1*-*GLI1*. **a** Chest X-ray and **b** computed tomography scan reveal an intrathoracic tumor in the left upper lesion (yellow arrows). **c** Hematoxylin-eosin staining shows malignant epithelioid neoplasm mimicking a neuroendocrine tumor. **d** The tumor is positive for CD56 by immunostaining. **e** Structure of the *PTCH1*-*GLI1* fusion gene. The dotted line indicates breakpoints. **f** Predicted domain structure of the PTCH1-GLI1 protein. The dotted line indicates breakpoints. **g** The tumor is positive for GLI1 by immunostaining. **h** Comparison of *GLI1* gene expression level between unique patient number (UPN) 36 and other patients. The *GLI1* expression level is significantly higher in UPN 36 than in other patients. **i** Volcano plot comparing the expression profiles between mock-transfected and *PTCH1*-*GLI1*-transfected HEK293T cells. *GLI1*, *PTCH1*, and *SOX18* expression levels are significantly higher in *PTCH1*-*GLI1* transfected cells. SNAG, SNAG domain; SSD, sterol-sensing domain; FPKM, fragments per kilobase of exon per million reads mapped.

We identified *PTCH-GLI1* fusion in an intrathoracic tumor from a 13-year-old boy (Fig. 2a and b). Computed tomography (CT)-guided tumor biopsy specimen showed small round cells with hyperchromasia forming an alveolar pattern by hematoxylin-eosin staining. He was tentatively diagnosed with ES based on immunostaining results showing that the tumor was negative for desmin and positive for CD56 and CD99. The tumor was resistant to standard ES chemotherapy, which comprised vincristine, doxorubicin, cyclophosphamide, ifosfamide, and etoposide. An additional tumor biopsy was performed six months after the first biopsy, and the histopathological diagnosis was revised to extraskeletal myxoid chondrosarcoma based on the clinical course; immunostaining, which was positive for vimentin, CD56, and S-100; and FISH examination, which was negative for the *EWSR1* split signal. He received 74 Gy heavy-particle radiotherapy and survived without tumor recurrence for seven years after treatment completion at last follow-up.

The histopathological sections from the second biopsy showed a small round-cell tumor comprising cells with uniform nucleus and cytoplasm arranged in a cord-like pattern (Fig. 2c). Immunostaining was positive for vimentin, CD56 (Fig. 2d), and S-100 and negative for chromogranin A, synaptophysin, CD34, α-smooth muscle actin, desmin, epithelial membrane antigen, and cytokeratin AE1/3. The sections were retrospectively reviewed by K.Y., M.N., and A.N., who reached the histopathological diagnosis of undifferentiated sarcoma.

The RNA-seq analysis detected *PTCH1*-*GLI1* fusion (Fig. 2e), generated by the fusion of exon 1a of *PTCH1* and exons 6–10 of *GLI1*. The predicted protein comprised 929 amino acids and contained the conserved functional zinc finger domains of GLI1. Interestingly, both *PTCH1* and *GLI1* are associated with the Sonic hedgehog (Shh) signaling pathway, in which *PTCH1* is a membrane receptor for ligands and *GLI1* is a transcription factor^19^. In addition, the 5′ untranslated region in exon 1a of *PTCH1* includes a binding site for GLI1^20^, suggesting the formation of an autonomous, positive-feedback loop mediated by the *PTCH1-GLI1* interaction. Thus, the presumed function of the *PTCH1*-*GLI1* fusion was upregulation of its expression and Shh pathway activation, which is associated with cell proliferation, apoptosis, angiogenesis, epithelial-to-mesenchymal transition, and stem cell self-renewal^21^ (Fig. 2f).

To confirm Shh signaling pathway activation in the tumor tissue, we analyzed *GLI1* gene expression using RNA-seq and confirmed that *GLI1* mRNA was expressed at a significantly higher level compared with other analyzed samples (Fig. 2h). Furthermore, immunostaining of the histopathological sections showed strong GLI1 positivity in the nucleus (Fig. 2g). In addition, to confirm the activation of Shh signaling pathway by the *PTCH1*-*GLI1* fusion gene, we overexpressed *PTCH1*-*GLI1* in HEK293T cells (CRL-1573, Funakoshi, Tokyo, Japan) (Fig. 2i). The top three upregulated genes were endogenous *GLI1*, endogenous *PTCH1*, and *SOX18*, all of which are part of the Shh signaling pathway and are targets of *GLI1* as a transcription factor^19,22^. These results suggested that *PTCH1-GLI1* fusion activated the Shh signaling pathway.

### SMARCA4-deficient undifferentiated sarcoma with *SMARCA4* alterations: UPN 66

**Fig. 3:**
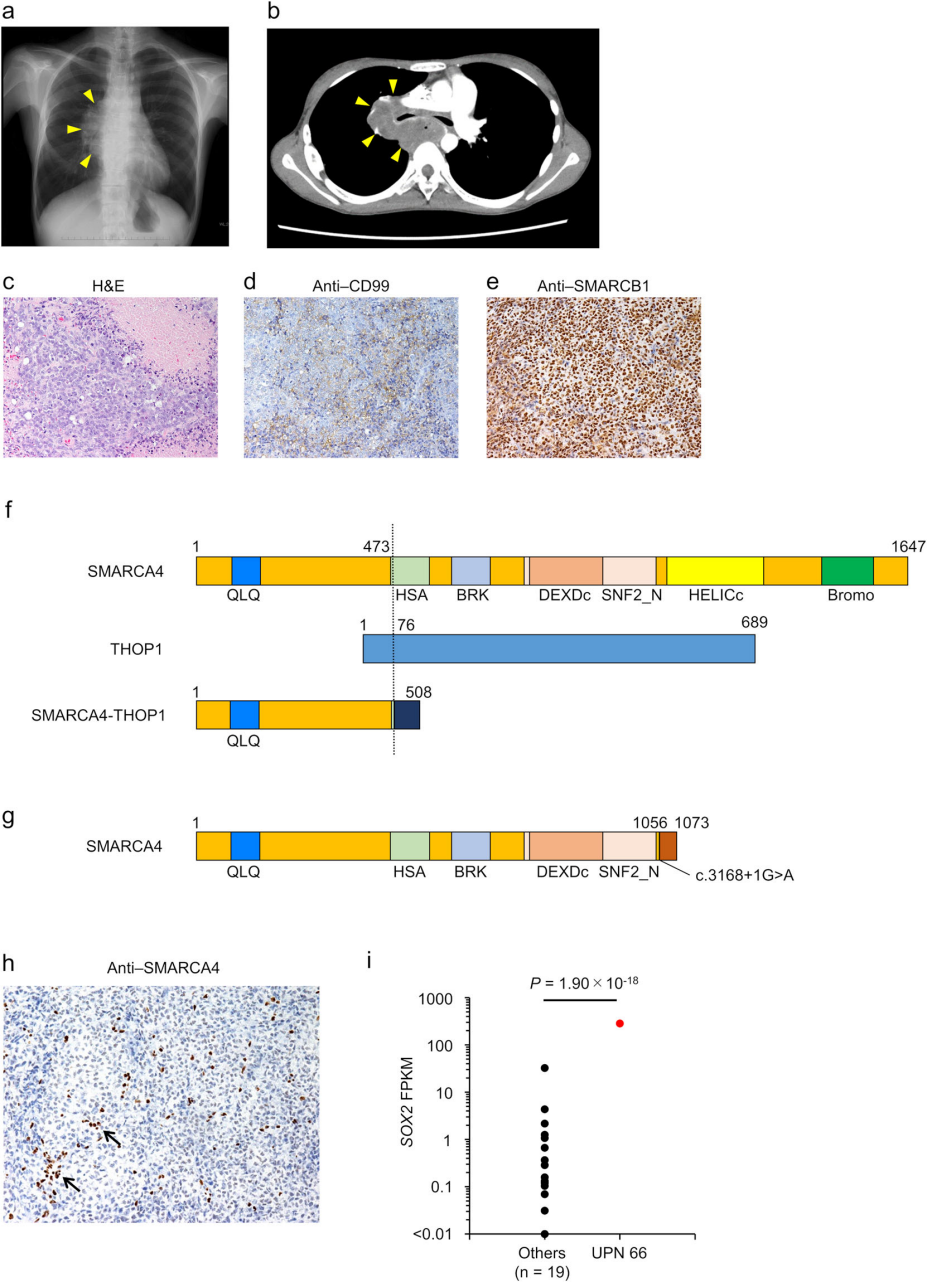
SMARCA4-deficient undifferentiated sarcoma with *SMARCA4*-*THOP1*. **a** Chest X-ray and **b** computed tomography scan reveal a tumor in the mediastinum and right lung (yellow arrow). **c** Hematoxylin-eosin staining reveals the proliferation of epithelioid cells with hyperchromatic or vesicular nuclei. **d** The tumor is positive for CD99 and **e** SMARCB1 by immunostaining. **f**, **g** Predicted domain structures of SMARCA4-THOP1 protein and SMARCA4 with point mutation. Dotted lines indicate the breakpoints. These proteins lack functional SMARCA4 domains. **h** By immunostaining, the tumor is negative for SMARCA4. Note that the lymphocytes are positive for SMARCA4 (black arrows). **i** Comparison of *SOX2* gene expression level between UPN 66 and other patients, showing that the *SOX2* expression level is significantly higher in UPN 66 compared to other patients. QLQ, Gln, Leu, Gln motif; HSA, helicase/SANT-associated domain; BRK, Brahma and Kismet domain; DEXDc, DEAD-like helicase superfamily domain; SNF2_N, SNF2 family N-terminal domain; HELICc, helicase superfamily C-terminal domain; Bromo, bromodomain.

We identified *SMARCA4* inactivating alterations in an undifferentiated sarcoma in a 15-year-old girl. She had a history of congenital esophageal atresia and received radical surgery. After surgery, she developed gastroesophageal reflux disease and was admitted after developing swallowing difficulty and vomiting. Chest CT scan revealed a large tumor in the mediastinum, which invaded the hilum of both lungs (Fig. 3a and b), and metastatic lesions were observed in the pleura and upper right supraclavicular region. Biopsy of the metastatic lymph node revealed proliferation of epithelioid cells with hyperchromatic or vesicular nuclei that were arranged in a sheet-like pattern, with limited necrosis in the background (Fig. 3c). Based on these pathological findings, the institutional diagnosis was anaplastic large-cell lymphoma. She received multi-drug chemotherapy based on the ALCL99 study regimen^23^ and a regimen comprising ifosfamide, carboplatin, and etoposide; however, she died due to disease progression. Immunostaining was positive for CD99 and SMARCB1 and negative for anaplastic lymphoma kinase, desmin, and myogenin (Fig. 3d and e). The histopathological diagnosis was undifferentiated sarcoma.

RNA-seq identified the presence of *SMARCA4*-*THOP1* fusion gene (Fig. 3f) comprising an out-of-frame combination between exons 1–8 of *SMARCA4* and exons 3–13 of *THOP1*. This fusion truncated the helicase ATP-binding and helicase C-terminal domains, the main functional domains of SMARCA4, strongly suggesting the loss-of-function with this fusion gene. In addition to the *SMARCA4*-*THOP1* fusion gene, RNA-seq also identified an *SMARCA4* splice-site mutation (c.3168+1G>A) (Fig. 3g), suggesting the diagnosis of SMARCA4-deficient undifferentiated sarcoma caused by biallelic inactivation of SMARCA4^24^. Additional immunostaining for SMARCA4 using a rabbit monoclonal antibody that recognizes the N-terminal region of the protein confirmed the complete loss of SMARCA4 expression (Fig. 3h), supporting the RNA-seq findings. Moreover, the tumor tissue overexpressed *SOX2*, a characteristic finding of SMARCA4-deficient neoplasms (Fig. 3i). Clinicopathologically, the patient was 15-year-old girl who had no history of smoking, and p53 expression was weak in the tumor cells by immunohistochemistry. So, the tumor was considered as malignant rhabdoid tumor rather than SMARCA4-deficient thoracic sarcoma^25^. Malignant rhabdoid tumors are associated with germline mutations of *SMARCB1* or *SMARCA4*, whereas no *SMARCA4* germline mutations have been reported in SMARCA4-deficient thoracic sarcomas.

Although peripheral blood mononuclear cells from the patient and her parents did not harbor any genetic *SMARCA4* alterations, an experienced clinical geneticist (S.M.) identified that the patient harbored dysmorphic features including epicanthus, deformed and low-set ears, thick lips, slender and curved nails, and slender toes, indicating Coffin-Siris syndrome with somatic mosaicism of *SMARCA4* mutation. In this patient, somatic mosaicism of either c.3168+1 G>A mutation or *SMARCA4-THOP1* fusion, followed by somatic acquisition of the other mutation, may have resulted in biallelic SMARCA4 inactivation. However, further investigation could not be performed due to the lack of clinical specimens other than the tumor specimens and peripheral blood mononuclear cells.

### Spindle cell rhabdomyosarcoma with *SRF-NCOA1* fusion: UPN 7

**Fig. 4:**
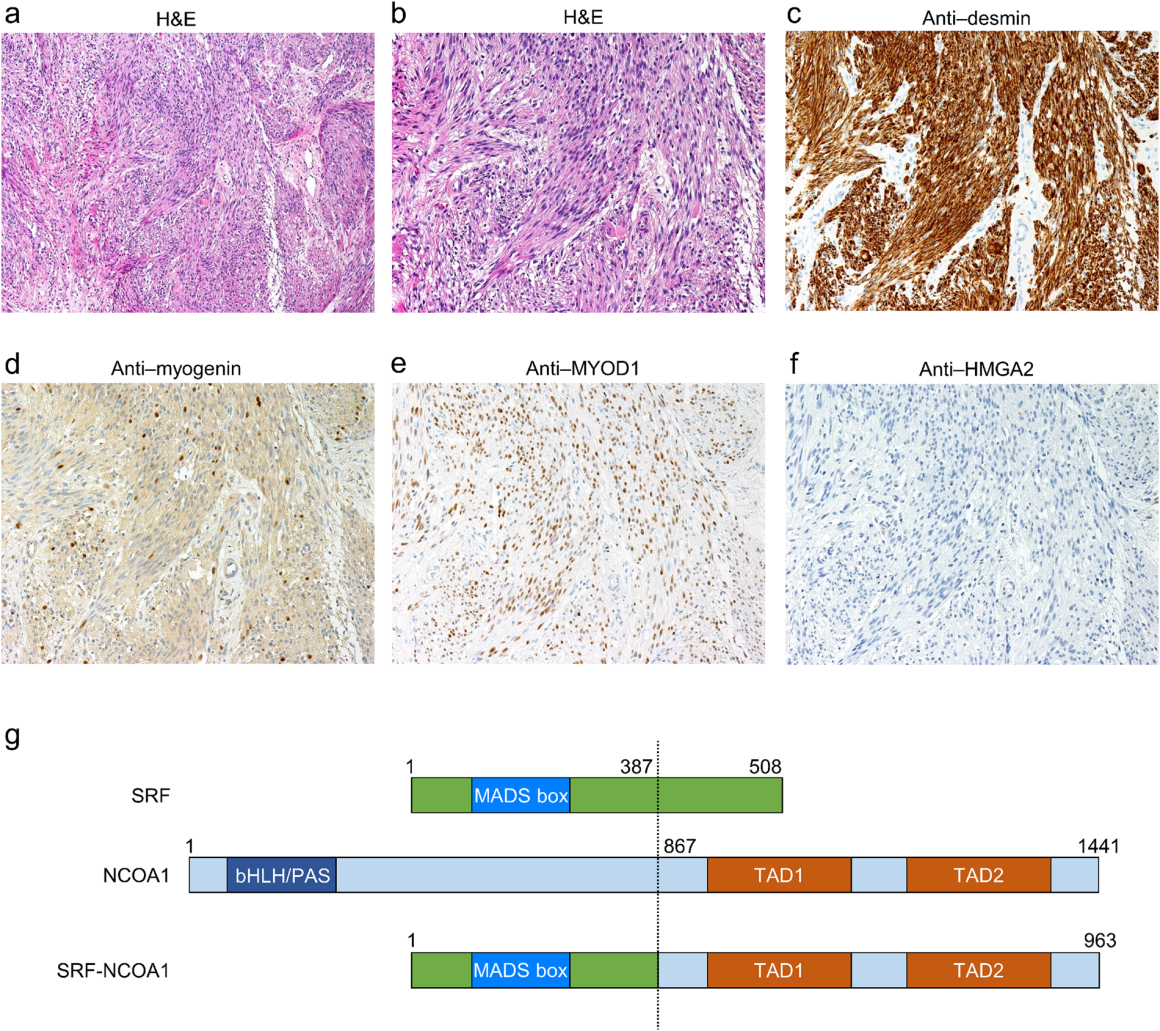
Spindle cell rhabdomyosarcoma with *SRF*-*NCOA1*. **a**–**f** Histopathological features of UPN 7. Hematoxylin-eosin staining shows spindle cell morphology. The tumor is strongly positive for desmin by immunostaining. The tumor is also positive for myogenin and MYOD1 but is negative for HMGA2 by immunostaining. **g** Domain structure of the SRF-NCOA1 protein. bHLH/PAS, basic helix-loop-helix-PER-ARNT-SIM; TAD, transactivation domain.

A 3-month-old girl developed a tumor on the left back, which was initially noticed at one month of age. The tumor biopsy section showed bundles of spindle cells; however, cellular atypism and mitotic figures were not detected. She was diagnosed with infantile fibromatosis and received three courses of chemotherapy with vincristine, actinomycin-D, and ifosfamide. The tumor size gradually decreased but showed regrowth after the completion of therapy, and a second biopsy was performed. Based on positive myogenin immunostaining, the pathological diagnosis was revised to rhabdomyosarcoma although the subtype was not identified. She received a total of 29 courses of chemotherapy, including the vincristine, actinomycin-D, and ifosfamide; vincristine and actinomycin-D; and vincristine, actinomycin-D, and cyclophosphamide regimens. The remaining tumor was surgically resected after therapy completion. She survived for ten years without tumor recurrence. The re-evaluation confirmed that the histopathological specimen showed spindle-like morphology (Figs. 4a and b) and was positive for desmin, myogenin, and myogenic differentiation 1 (MYOD1) (Fig. 4c–e) and negative for high-mobility group AT-hook 2 (HMGA2) (Fig. 4f), which is expressed primarily in *PAX3/7-FOXO1*-negative rhabdomyosarcoma^26^. These histopathological findings were consistent with the diagnosis of spindle cell rhabdomyosarcoma.

We identified the presence of *SRF*-*NCOA1* fusion gene, which was formed by the in-frame combination of exons 1–4 of *SRF* with exons 13–21 of *NCOA1*. The predicted protein comprising 963 amino acids retained an SRF-derived MADS-box domain and NCOA1-derived transactivation domains (Fig. 4g). The *SRF-NCOA1* fusion was recently reported in well-differentiated rhabdomyosarcoma^18^, and the *SRF*-*NCOA2* fusion gene formed by the in-frame fusion of exons 1–6 of *SRF* and exons 12–22 of *NCOA2* was previously reported in congenital/infantile spindle cell rhabdomyosarcoma^27^. The predicted SRF-NCOA2 protein retains the SRF MADS-box domain and the NCOA2 transactivation domains, similar to the predicted protein structure for SRF-NCOA1. The similarities in the clinicopathologic features between the previously reported cases and our patient suggested that patients with *SRF-NCOA1/2* fusion genes might comprise a unique rhabdomyosarcoma subtype.

### Gene expression-based clustering

We performed gene expression-based clustering including 20 patients with samples available for extraction of RNA with sufficient quality for the RNA-seq using the poly (A) capture method. The unsupervised clustering analysis identified two distinct patient groups. Cluster 1 (*n* = 10) comprised nine patients with rhabdomyosarcoma (alveolar [*n* = 3], embryonal [*n* = 4], and sclerosing [*n* = 2]) and one patient with fetal rhabdomyomatous nephroblastoma (UPN 57). Cluster 2 (*n* = 10) comprised four patients with ES, three patients each with undifferentiated sarcoma, and other tumor types (Supplementary Fig. 5a).

Differential gene expression analysis comparing Clusters 1 and 2 revealed that Cluster 1 was characterized by the overexpression of *MYOG and CHRNG* (Supplementary Fig. 5b), which are sensitive and specific genetic markers for rhabdomyosarcoma^28,29^. Gene-set enrichment analysis revealed the enrichment of *PAX3*-*FOXO1*-associated genes in Cluster 1 (GRYDER_PAX3FOXO1_TOP_ENHANCERS, Supplementary Fig. 5c)^30^, whereas Cluster 2, which included ES associated with *EWSR1-FLI1* fusion, undifferentiated sarcoma, and other tumors, was not associated with specific highly expressed genes (Supplementary Fig. 5b). Further assessment of the expression levels of several genes associated with the gene set revealed that all ten patients, including the patient with rhabdomyomatous nephroblastoma (UPN 57), in Cluster 1 exhibited higher *MYOG* and *MYOD1* expression levels compared to those in Cluster 2 (Supplementary Fig. 5d).

The patient diagnosed with fetal rhabdomyomatous nephroblastoma (UPN 57) was included in Cluster 1, along with the patients with rhabdomyosarcoma. The overexpression of *MYOD1* and *CHRNG* observed in this patient with fetal rhabdomyomatous nephroblastoma could explain their inclusion in Cluster 1 (Supplementary Fig. 5a, d, and e). The histopathological re-evaluation of the case showed that presence of blastemal, epithelial, and mesenchymal rhabdomyomatous cells by hematoxylin-eosin staining. Immunostaining showed that the rhabdomyomatous cells were positive for desmin, myogenin, and paired box 2 and negative for Wilms tumor 1 (Supplementary Fig. 5f–k).

## Discussion

Molecular assessment to determine the presence of the recently identified genetic events in pediatric tumors has been shown to be a promising approach in improving and refining diagnosis. We performed a pilot study using RNA-seq to evaluate 47 patients with suspected sarcomas and identified genetic events in 23 (49%) of the cases.

Undifferentiated sarcoma shows no identifiable marker of differentiation according to the analyses based on currently available technology^31^ and is defined as a heterogenous group, with diagnosis based on exclusion, although genetic subgroups, such as *BCOR*-rearranged tumors^10^ and *CIC*-rearranged tumors^11^, have recently been defined. In the current study, 5 of the 47 patients were diagnosed with undifferentiated sarcoma by consensus histopathological review, including FISH analysis based on the preference of pathologist; four of these five cases (80%) could be more accurately subcategorized based on the detection of fusion genes by RNA-seq (Fig. 1). Therefore, RNA-seq may be particularly useful in the diagnosis and subcategorization of undifferentiated sarcoma. Moreover, five other patients (11%) patients received additional information related to their prognosis based on the identification of genetic events. These findings support the clinical value of RNA-seq analysis during the diagnostic process for pediatric patients with suspected sarcomas. However, this was a small pilot study and the patient selection bias could not be denied because the study was limited to cases with available frozen tumor specimens.

The *PTCH1*-*GLI1* fusion gene, which was detected in one patient with undifferentiated sarcoma, was previously reported in a case of malignant epithelioid neoplasm^17^. Our additional functional analyses showed that this specific *PTCH1*-*GLI1* fusion activated the Shh signaling pathway through an auto-feedback mechanism. Shh signaling regulates stem cell homeostasis in adult tissues, whereas persistent Shh pathway activation leads to various cancers, including basal cell carcinoma and medulloblastoma^32^. Other *GLI1*-rearranged fusion genes that have been reported to be associated with tumors include *ACTB*-*GLI1* in pericytoma^33^ and *MALAT1*-*GLI1* in gastroblastoma and plexiform fibromyxoma^34,35^. In these fusion genes, GLI1 is overexpressed because *ACTB* and *MALAT1* act as strong promoters^33,35^. Therefore, it is likely that these *GLI1*-rearranged fusion genes activate Shh signaling through the increased expression of GLI1, supporting the involvement of *PTCH1*-*GLI1* fusion in tumor development.

With recent developments in molecular targeted therapies such as tyrosine kinase inhibitors, proteasome inhibitors, antiapoptotic Bcl-2 inhibitors, and monoclonal antibodies, RNA-seq analysis may also be useful in improving treatment strategies and outcomes in pediatric solid tumors. A potential example in our case series is the demonstration that the Shh signaling pathway was activated in *GLI1*-rearranged sarcoma, which may be sensitive to Shh pathway inhibitors^21^. Moreover, in the present study the integrated diagnosis using RNA-seq was useful for clinical management, especially for determining chemotherapy regimens, in some of the recently diagnosed patients, including those diagnosed with SMARCA4-deficient undifferentiated sarcoma, NUT carcinoma, alveolar rhabdomyosarcoma, and synovial sarcoma.

In both spindle cell and sclerosing rhabdomyosarcoma, the identified fusion genes included rearrangements in either *VGLL* or *NCOA* and the *MYOD1* p.L122R mutation was observed^36^. The fusion genes, which were detected in infants, were associated with a favorable clinical course^36^. In contrast, the *MYOD1* mutation was detected in older children and was associated with a highly aggressive behavior^36^. These results suggested that employing molecular approaches are important for determining effective treatment strategies because the prognosis often differs depending on the type of genetic alteration even in cases with similar pathological diagnosis.

Furthermore, in the present study, we detected the novel *SMARCA4-THOP1* fusion gene, which causes SMARCA4 biallelic inactivation, together with a point mutation in SMARCA4-deficient undifferentiated sarcoma.

Our unsupervised clustering analysis based on gene expression profiles led to the identification of a cluster that primarily comprised patients with rhabdomyosarcoma. Importantly, *MYOG* and *CHRNG* were overexpressed in this cluster, indicating that referencing gene expression profiles may assist with the categorization and diagnosis of pediatric sarcomas. Furthermore, performing gene expression analysis may be useful during the diagnosis process primarily for patients without a clear diagnostic genetic event. The number of patients in the present study was small; therefore, we could not perform sufficient analyses for other tumor types and the identification of additional diagnostic clusters requires cluster analysis of larger patient cohorts in future studies.

In conclusion, RNA-seq-based genetic analysis can aid in the histopathological diagnosis and the development of stratified treatment strategies for pediatric patients with suspected sarcomas.

## Methods

### Patients

Among a total of 88 consecutive pediatric patients with suspected sarcoma at initial diagnosis who were treated at the Nagoya University Hospital, 47 (54%) patients with available frozen tissue specimens were included in the present study. The diagnoses of the entire cohort (*n* = 88) and the analyzed patients (*n* = 47) are presented in Supplementary Table 3. The median age at diagnosis was 70 (range, 1–215) months. The institutional histologic diagnoses were recorded for all cases. Written informed consent was obtained from all patients or their parents. The study was approved by the Ethics Committee of the Nagoya University Graduate School of Medicine and was conducted in accordance with the principals of the Declaration of Helsinki.

### Samples

Fresh-frozen tissues obtained by biopsy or tumorectomy were used for DNA and RNA extraction. Genomic DNA was extracted using the QIAamp DNA Blood Mini Kit (QIAGEN, Hilden, Germany), according to the manufacturer’s instructions. To extract total RNA, the frozen samples were homogenized with a mortar and pestle in the presence of liquid nitrogen. The homogenized samples were collected using Buffer RLT with 0.14 M 2-mercaptoethanol, and RNA was extracted using the RNeasy Mini Kit (QIAGEN). The RNA integrity score (RNA integrity number equivalent; RINe) was assessed using RNA ScreenTape with the TapeStation 2200 system (Agilent, Santa Clara, CA, USA).

### Histopathological examination

First, the initial diagnosis, i.e., institutional diagnosis, was described based on histopathological examination reports at disease onset. Next, histopathological re-examination was performed to reach a consensus diagnosis without RNA-seq by histopathologists who specialized in pediatric solid tumors (A.N., M.N., and K.Y.), following an institutional diagnostic flow-chart for pediatric solid tumors (Supplementary Fig. 1). Examinations were based on hematoxylin-eosin staining of formalin-fixed, paraffin-embedded tissue samples and immunostaining using antibodies against MYOD1 (dilution 1:50; 5.8 A, Dako, Heverlee, Belgium), desmin (dilution 1:100; D33, Dako), myogenin (dilution 1:100; F5D, Dako), HMGA2 (dilution 1:100; rabbit polyclonal, ab97276, Abcam, Cambridge, MA, USA), CD99 (dilution 1:800; O13, Thermo Fisher Scientific, Waltham, MA, USA), NKX2.2 (dilution 1:1000; 74.5A5, BD Pharmingen, San Jose, CA, USA), CCNB3 (dilution 1:50; rabbit polyclonal, HPA000496, Sigma-Aldrich, St. Louis, MO, USA), CD56 (dilution 1:100; 1B6, Leica Biosystems, Buffalo Grove, IL, USA), GLI1 (dilution 1:600; rabbit polyclonal, NB600-600, NOVUS, Centennial, CO, USA), SMARCA4 (dilution 1:100; EPNCIR111A, Abcam), SOX2 (dilution 1:500; sc-365964, Santa Cruz Biotechnology, Dallas, TX, USA), NUTM1 (dilution 1:50; C52B1, Cell Signaling, Danvers, MA, USA), PAX2 (dilution 1:100; rabbit polyclonal, 21385-1-AP, Proteintech, Rosemont, IL, USA), and WT1 (dilution 1:20; WT49, Leica Biosystems). Other antibodies used in the present study are listed in Supplementary Fig. 1. FISH was performed as necessary using a Vysis LSI EWSR1 (22q12) dual-color, break-apart rearrangement probe (07J71-001, Abbott, Abbott Park, IL, USA), a Vysis FOXO1 break-apart FISH probe (03N60-020; Abbott), a *FOXO1/PAX3* dual-color, single-fusion probe (Z2018, ZytoVision, Bremerhaven, Germany), and a *FOXO1/PAX7* dual-color, single-fusion probe (Z2019, ZytoVision), when the specimen amount was sufficient.

### RNA-sequencing

Non-directional sequencing libraries were prepared using the NEBNext Ultra RNA Prep Kit for Illumina and the NEBNext Poly(A) mRNA Magnetic Isolation Module (for RNA with RINe ≥7.0) or the NEBNext rRNA Depletion Kit (for RNA with RINe <7.0) (New England Biolabs, Ipswich, MA), according to the manufacturer’s instructions. Prepared libraries were run on a HiSeq 2500 next-generation sequencing (NGS) platform, with 2 × 75 bp paired-end reads (Illumina, San Diego, CA). The obtained reads were aligned with the hg19 human reference sequence using Tophat2 (version 2.0.14) and its fusion search option (TopHat-Fusion) for the detection of fusion genes^37,38^. Using the default parameters, candidate fusion genes supported by five or more reads were called and fusions and fusion partner genes already known to be a cause of sarcoma were searched. In addition, a manual search was performed for novel fusion gene candidates with more than 30 reads using the Integrative Genomics Viewer (https://software.broadinstitute.org/software/igv/home). To confirm the presence of candidate fusion genes, Sanger sequencing was performed for RT-PCR products obtained by the SuperScript III reverse transcription system (Life Technologies, Carlsbad, CA, USA) and PrimeSTAR GXL DNA Polymerase (TaKaRa Bio, Ohtsu, Japan). The primers for RT-PCR were shown in Supplementary Table 5.

TopHat2-generated.bam files were analyzed by VarScan2 (version 2.3.6)^39^ to identify candidate single nucleotide variants with a variant allele frequency >0.1 (10%) for 12 genes (Supplementary Table 2) that are previously reported to be diagnostic in specific sarcoma subtypes, which were annotated by ANNOVAR (version 2016-02-01)^40^. Candidate gene variants were confirmed by Sanger sequencing.

Fragments per kilobase of exon per million reads mapped (FPKM) values were calculated using Cufflinks (version 2.2.1)^41^. Differential expression analysis was performed using HTSeq (version 0.6.1)^42^ and DESeq2 (version 1.30.0)^43^ with default parameters. Cluster 3.0 was used to group samples based on hierarchical clustering with the average linkage method^44^. Briefly, raw count data for each gene, obtained by RNA-seq, were normalized by variance-stabilizing transformation, using DESeq2, and the values were adjusted to center the genes relative to the medians. Finally, clustering analysis was performed with the RNA-seq data of 20 patients who were analyzed by the poly (A) capture method using Pearson’s correlation coefficient analysis, because different RNA-seq sample preparation methods can lead to large differences in gene expression profiling data. Results were visualized using Java TreeView^45^.

### Exogenous expression of PTCH1-GLI1

The complete coding sequence of *PTCH1*-*GLI1* was cloned into CSIV-CMV-MCS-IRES2-Venus, a self-inactivating lentiviral vector construct which was a generous gift from Dr. Hiroki Miyoshi (Riken BioResource Center). HEK293T cells were transfected with the plasmid including *PTCH1*-*GLI1* or the mock vector using ScreenFect™A (Wako Pure Chemical Industries, Osaka, Japan). The medium was removed 16 h later, and the cells were incubated with complete RPMI1640 medium supplemented with 10% fetal bovine serum. Forty-eight hours post-transfection, the cells were sorted using Venus fluorescence with a FACSAria II Cell Sorter (BD Biosciences, Franklin Lakes, NJ) for global expression analysis.

### Statistical analysis

The *t*-test was used to compare gene expression levels between disease groups. All *P* values reported are two-sided, and *P* values < 0.05 were considered significant. All statistical analyses were performed using EZR (Saitama Medical Center, Jichi Medical University), a graphical user interface for R (The R Foundation for Statistical Computing, Vienna, Austria)^46^.

### Reporting summary

Further information on research design is available in the Nature Research Reporting Summary linked to this article.

## Supplementary information

Supplementary Information

Reporting Summary

## Data Availability

Sequence data have been deposited at the DNA Data Bank of Japan (DDBJ) Japanese Genotype-phenotype Archive, under accession number JGAS000284.
