# Supplementary material for: Integrated diagnosis based on transcriptome analysis in suspected pediatric sarcomas
Source: NPJ Genom Med. 2021 Jun 15;6:49. doi: 10.1038/s41525-021-00210-y (PMC8206218; doi:10.1038/s41525-021-00210-y)
Supplement: Supplementary file 1 — Supplementary Information [file 41525_2021_210_MOESM1_ESM.pdf]

Supplementary Information

Integrated diagnosis based on transcriptome analysis in suspected pediatric sarcomas

Ichikawa, D. et al

Supplementary Notes **Page.3**

Case report of NUT carcinoma with *ZNF532-NUTM1* fusion: UPN 64

Case report of clear cell sarcoma of the kidney with *BCOR-CCNB3* fusion: UPN 42

Supplementary Figures **Page. 4–12**

Supplementary Figure 1. Diagnostic flowchart for consensus diagnosis

Supplementary Figure 2. Sanger sequence of candidate fusion genes and mutations

Supplementary Figure 3. NUT carcinoma, with *ZNF532-NUTM1*

Supplementary Figure 4. Clear cell sarcom of the kidney, with *BCOR-CCNB3*

Supplementary Figure 5. Gene expression-based clustering

Supplementary Tables **Page. 13–18**

Supplementary Table 1. Details of all patient diagnoses

Supplementary Table 2. List of genes for point mutation analysis

Supplementary Table 3. Institutional diagnosis of entire cohort and analyzed cohort

Supplementary Table 4. Details of discrepancy between institutional diagnosis and consensus diagnosis without RNA-seq information

Supplementary Table 5. List of primers for RT-PCR

Supplementary References **Page. 19**

Supplementary Notes

Case report of NUT carcinoma with ZNF532-NUTM1: UPN 64

We identified *ZNF532-NUTM1* fusion in a 1-year-old boy, who presented with abdominal pain, fever, and gait disturbance, accompanied by bladder and bowel dysfunction. Chest CT scan revealed a left pleural tumor, sized 5.0 cm × 6.0 cm, left lung atelectasis, left pleural effusion, and osteolytic bone lesion in the left rib **(Supplementary Fig. 3a and b)**. Fluorodeoxyglucose-positron emission tomography/CT (FDG-PET/CT) scan of the pleural tumor was FDG-positive **(Supplementary Fig. 3c)**. Magnetic resonance imaging showed spinal cord compression, caused by a metastatic lesion into the spinal canal.

During histopathological examination, diffuse, small circular cells, with distinct nucleoli and many nuclear fission events, were observed **(Supplementary Fig. 3d)**. Immunostaining was negative for desmin, myogenin, CD99, and NKX2.2 and positive for SMARCA4 and SMARCB1. He was diagnosed with undifferentiated sarcoma and treated with chemotherapy, which comprised vincristine, doxorubicin, cyclophosphamide, ifosfamide, and etoposide. His tumor showed a partial response to chemotherapy, and his neurological complications improved.

RNA-seq identified a *ZNF532-NUTM1* fusion, which contained *ZNF532* exon 1–8, a part of *ZNF532* intron 8, and *NUTM1* exon 4–7 **(Supplementary Fig. 3f)**. The predicted protein contains 1,997 amino acids, including the N-terminal 1,050 amino acids of ZNF532, which encodes 8 of 12 zinc finger domains **(Supplementary Fig. 3g)**. Additional immunostaining for NUTM1 showed the overexpression of NUTM1 **(Supplementary Fig. 3e)**.

Case report of clear cell sarcoma of the kidney with BCOR-CCNB3 fusion: UPN 42

We identified a *BCOR-CCNB3* fusion in a 14-year-old boy, who presented with abdominal pain, back pain, and gross hematuria. An abdominal CT scan revealed a tumor, sized 13 cm × 10 cm × 18 cm, in the left kidney and lymphadenopathy in the abdominal paraaortic area (**Supplementary Fig. 4a and b**). He received a laparoscopic tumorectomy and left nephrectomy. Histopathological examination revealed the diffuse proliferation of spindle-shaped tumor cells, with a branching capillary vasculature (**Supplementary Fig. 4c**). Immunostaining was negative for CD34, αSMA, CD99, and S-100 protein, and positive for SMARCB1. He was diagnosed with clear cell sarcoma of the kidney. Following surgery, he received 10.8 Gy local irradiation and 24 weeks of chemotherapy, based on the Japan Wilms Tumor Study Group-1 (JWiTS-1) protocol[1]. Four months after completing the treatment, his tumor recurred at multiple sites (abdominal cavity, retroperitoneal cavity, the abdominal paraaortic lymph nodes, and left lung). He received palliative chemotherapy and died of the disease 2 years after relapse.

We identified *BCOR-CCNB3* fusion by RNA-seq. *BCOR-CCNB3* consists of the in-frame fusion between *BCOR* exons 1–15 and *CCNB3* exons 5–12 (**Supplementary Fig. 4e**), consisting of 3,038 amino acids and containing the previously reported structures associated with this fusion gene[2]. This fusion gene contains all of the *BCOR* functional domains but lacks the *CCNB3* D-box domain (**Supplementary Fig. 4f**). Anti–CCNB3 immunostaining showed strong positive staining in the nucleus (**Supplementary Fig. 4d**).

Supplementary Figures

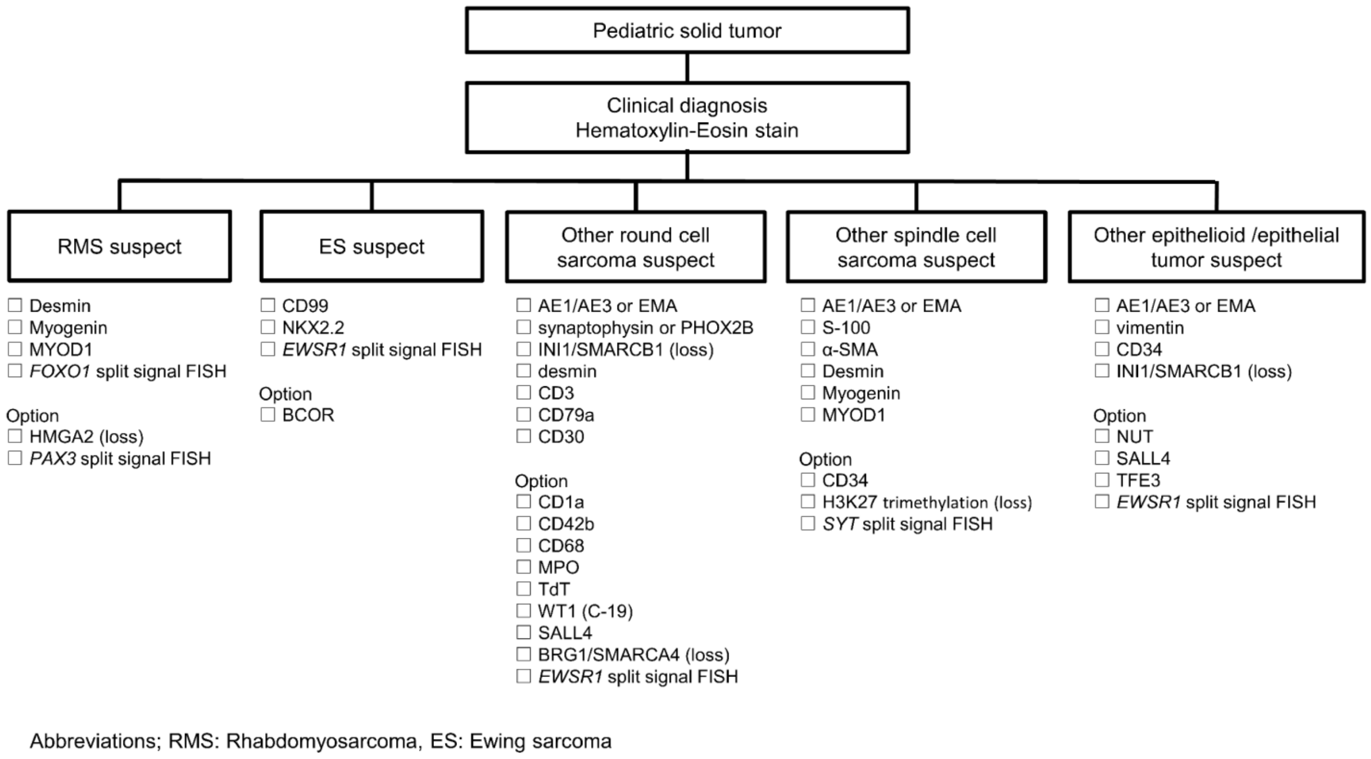

Supplementary Figure 1. Diagnostic flowchart for consensus diagnosis without RNA-seq information. Suggested flowchart for consensus diagnosis without RNA-seq information. Evaluation of hematoxylin and eosin (H&E) staining and clinical diagnosis can be used as the first step. Next, the section was divided into 5 groups, based on H&E staining, and characteristic immunostaining was performed for each group, as necessary. FISH analysis can also detect diagnostic fusion genes. If the section could not be categorized into a specific group based on H&E staining, we performed additional immunostaining or/and FISH, as necessary.

RMS: Rhabdomyosarcoma, ES: Ewing sarcoma, FISH; fluorescence in situ hybridization, MPO; Myeloperoxidase, TdT; Terminal deoxynucleotidyl transferase, SALL4; Sal-like protein 4, AE1; Anti-Cytokeratin 1, AE3; Anti-Cytokeratin 3, α-SMA; α-smooth muscle actin, TFE3; Transcription factor E3.

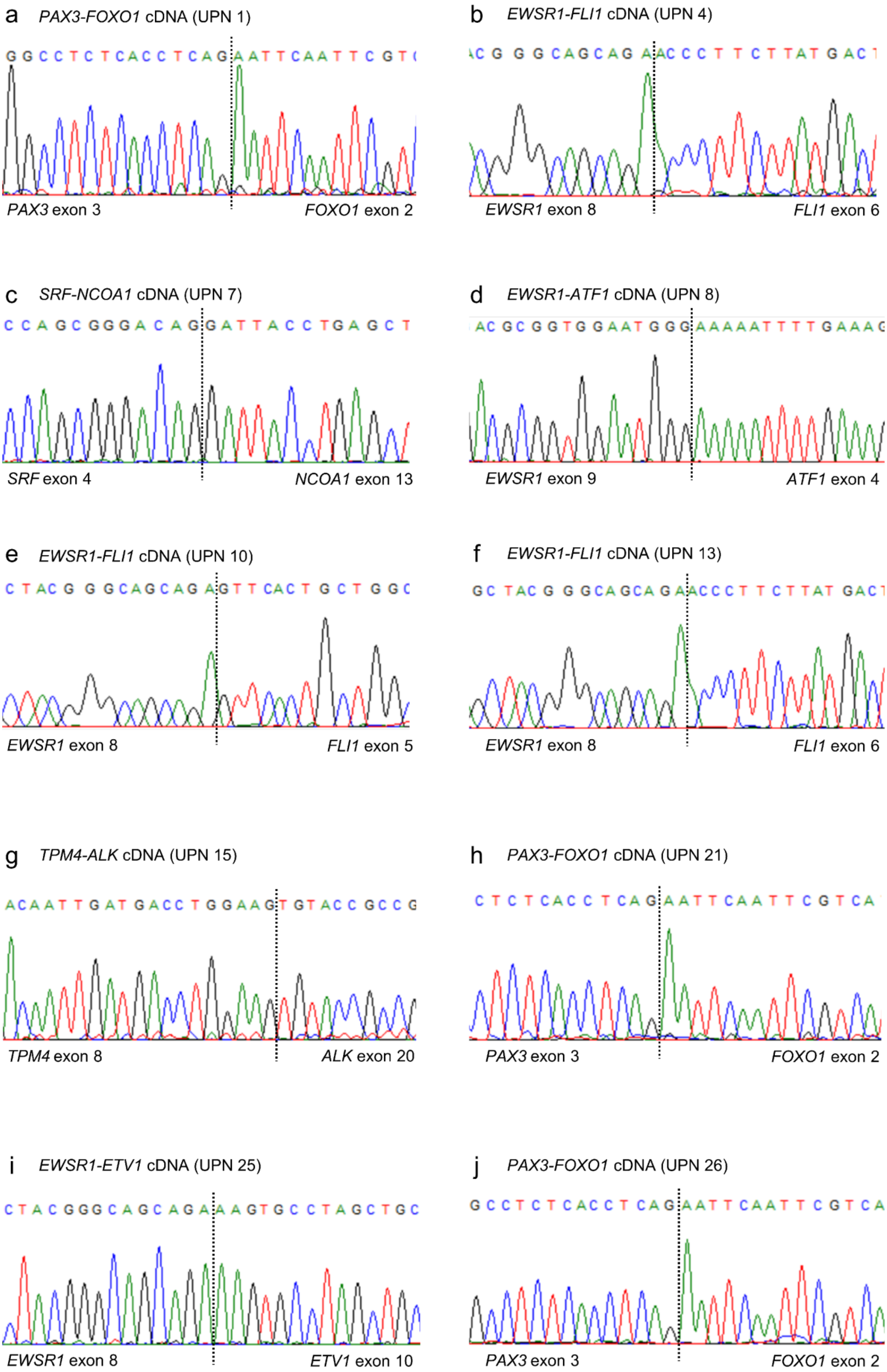
a
PAX3-FOXO1 cDNA (UPN 1)
GGCCTCTCACCTCAGAATTCAATTCGT
PAX3 exon 3
FOXO1 exon 2
b
EWSR1-FLI1 cDNA (UPN 4)
CGGGCAGCAGAACCCTTCTTATGAC
EWSR1 exon 8
FLI1 exon 6
c
SRF-NCOA1 cDNA (UPN 7)
CCAGCGGGACAGGATTACCTGAGCT
SRF exon 4
NCOA1 exon 13
d
EWSR1-ATF1 cDNA (UPN 8)
ACGCGGTGGAATGGGAAAAATTTTGAAA
EWSR1 exon 9
ATF1 exon 4
e
EWSR1-FLI1 cDNA (UPN 10)
CTACGGGCAGCAGAGTTCACTGCTGG
EWSR1 exon 8
FLI1 exon 5
f
EWSR1-FLI1 cDNA (UPN 13)
GCTACGGGCAGCAGAACCCTTCTTATGAC
EWSR1 exon 8
FLI1 exon 6
g
TPM4-ALK cDNA (UPN 15)
ACAATTGATGACCTGGAAGTGTACCGCCG
TPM4 exon 8
ALK exon 20
h
PAX3-FOXO1 cDNA (UPN 21)
CTCTCACCTCAGAATTCAATTCGTCA
PAX3 exon 3
FOXO1 exon 2
i
EWSR1-ETV1 cDNA (UPN 25)
CTACGGGCAGCAGAAAGTGCCTAGCTGC
EWSR1 exon 8
ETV1 exon 10
j
PAX3-FOXO1 cDNA (UPN 26)
GCCTCTCACCTCAGAATTCAATTCGTCA
PAX3 exon 3
FOXO1 exon 2

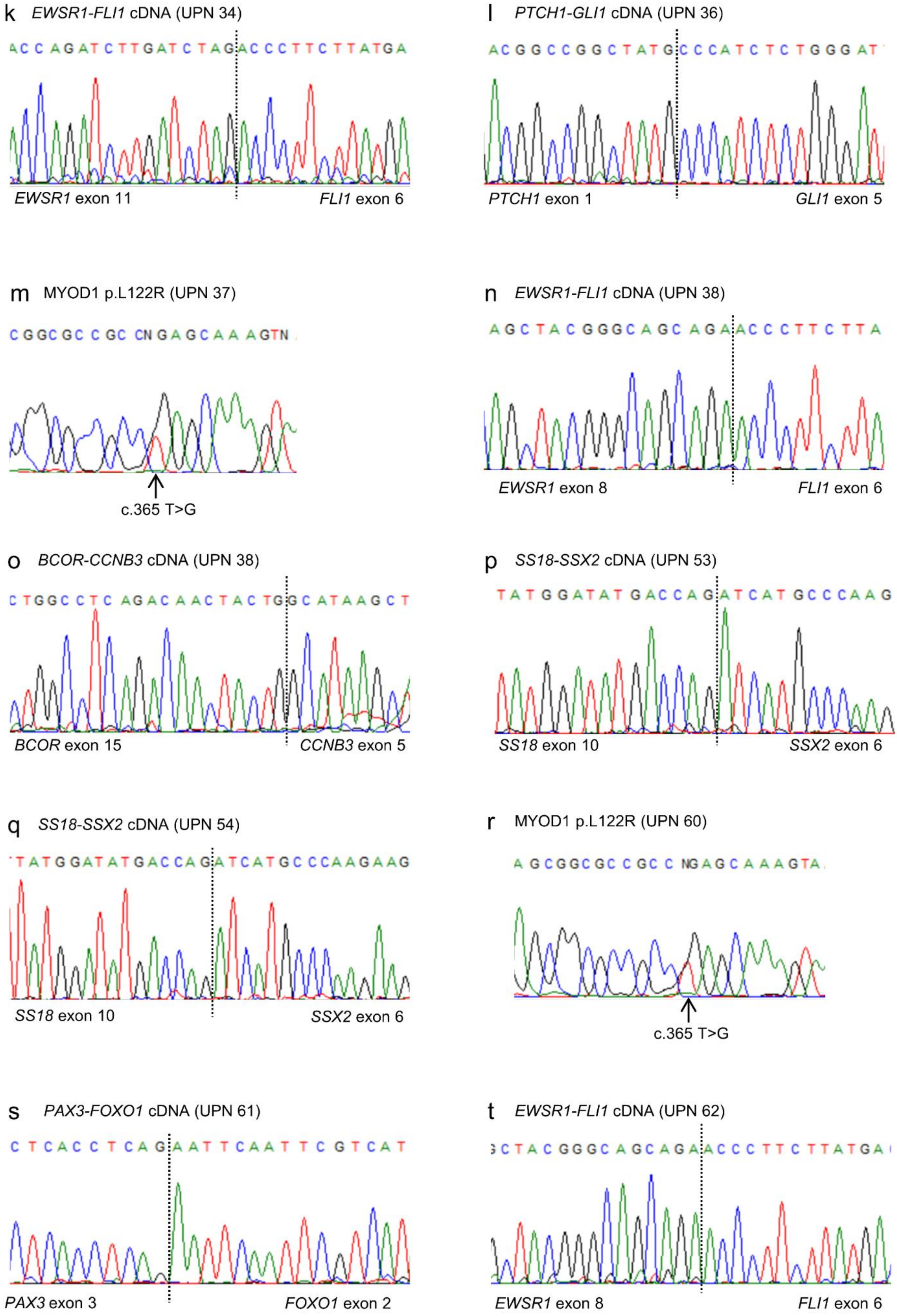
k EWSR1-FLI1 cDNA (UPN 34)
EWSR1 exon 11
FLI1 exon 6
l PTCH1-GLI1 cDNA (UPN 36)
PTCH1 exon 1
GLI1 exon 5
m MYOD1 p.L122R (UPN 37)
c.365 T>G
n EWSR1-FLI1 cDNA (UPN 38)
EWSR1 exon 8
FLI1 exon 6
o BCOR-CCNB3 cDNA (UPN 38)
BCOR exon 15
CCNB3 exon 5
p SS18-SSX2 cDNA (UPN 53)
SS18 exon 10
SSX2 exon 6
q SS18-SSX2 cDNA (UPN 54)
SS18 exon 10
SSX2 exon 6
r MYOD1 p.L122R (UPN 60)
c.365 T>G
s PAX3-FOXO1 cDNA (UPN 61)
PAX3 exon 3
FOXO1 exon 2
t EWSR1-FLI1 cDNA (UPN 62)
EWSR1 exon 8
FLI1 exon 6

u *PAX3-FOXO1* cDNA (UPN 63)

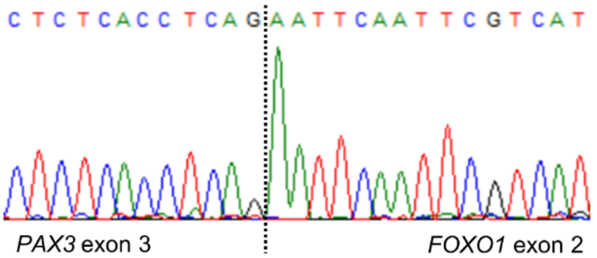

v *ZNF532-NUTM1* cDNA (UPN 64)

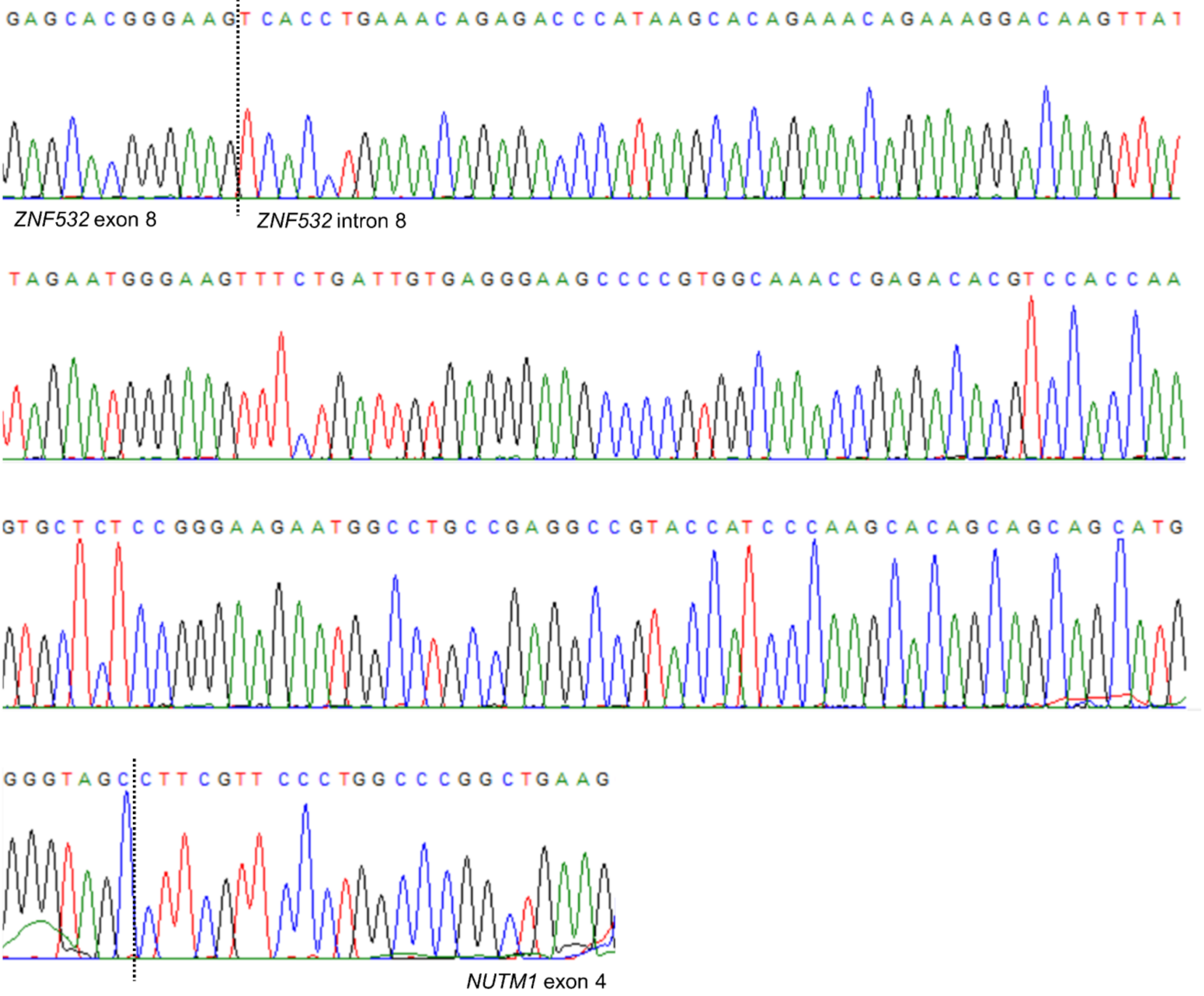

w *SMARCA4-THOP1* cDNA (UPN 66)

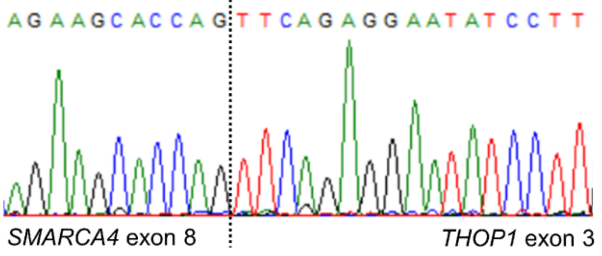

x *SMARCA4* gDNA (UPN 66)

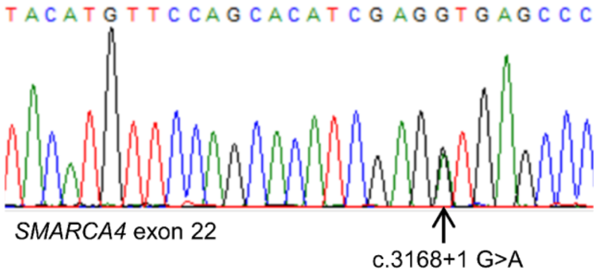

Supplementary Figure 2. Sanger sequence of candidate fusion genes and mutations. Candidate gene alterations obtained by RNA-seq were validated by Sanger sequence of RT-PCR product (**a–w**) or genomic DNA (**x**).

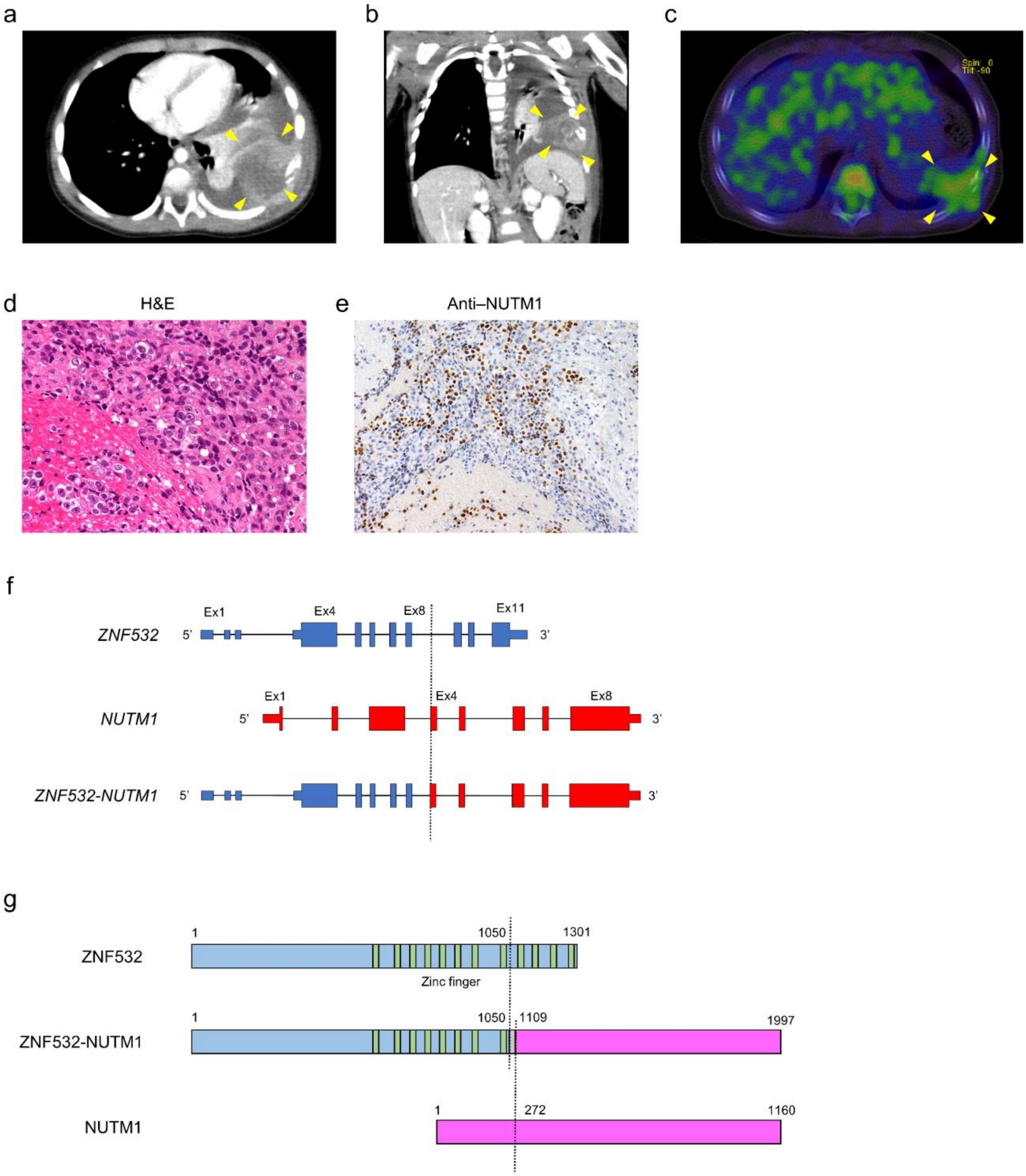

Supplementary Figure 3. NUT carcinoma with *ZNF532-NUTM1*. (**a** and **b**) CT scan revealed a tumor in the left pleural region (yellow arrow). (**c**) PET/CT scan revealed an FDG-positive pleural tumor. (**d**) Hematoxylin-eosin staining shows diffuse, small, circular cells, with distinct nucleoli and many nuclear fission events. (**e**) NUTM1 immunostaining was positive in the nucleus. (**f**) Genomic structure of the *ZNF532-NUTM1* fusion gene. Boxes correspond to exonic regions. Dotted lines indicate breakpoints. (**g**) Protein structure encoded by the *ZNF532-NUTM1* fusion gene.

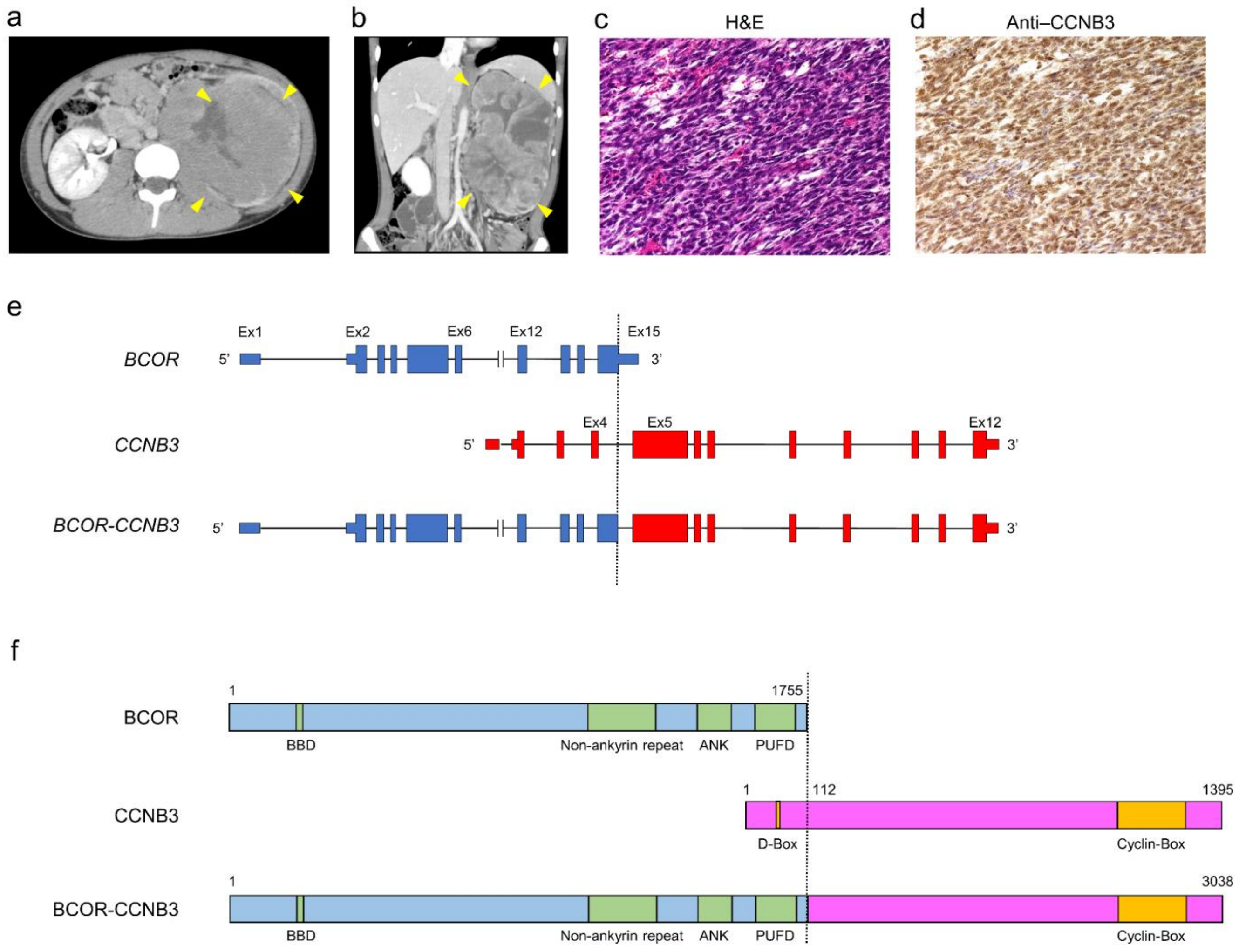

Supplementary Figure 4. Clear cell sarcoma of the kidney with *BCOR-CCNB3.* (**a** and **b**) CT scan revealed a tumor in the left kidney (yellow arrow). (**c**) Histopathological analysis revealed diffuse tumor cells, with a small, rounded or elliptical nucleus. (**d**) CCNB3 immunostaining was strongly positive in the nucleus. (**e**) Genomic structure of the *BCOR-CCNB3* fusion gene. Boxes correspond to exonic regions. Dotted lines indicate breakpoints. (**f**) Protein structures encoded by the *BCOR-CCNB3* fusion gene.

BBD: BCL-6 binding domain, ANK: Ankyrin repeat, PUFD: PCGF Ub-like fold discriminator, D-Box: destruction box.

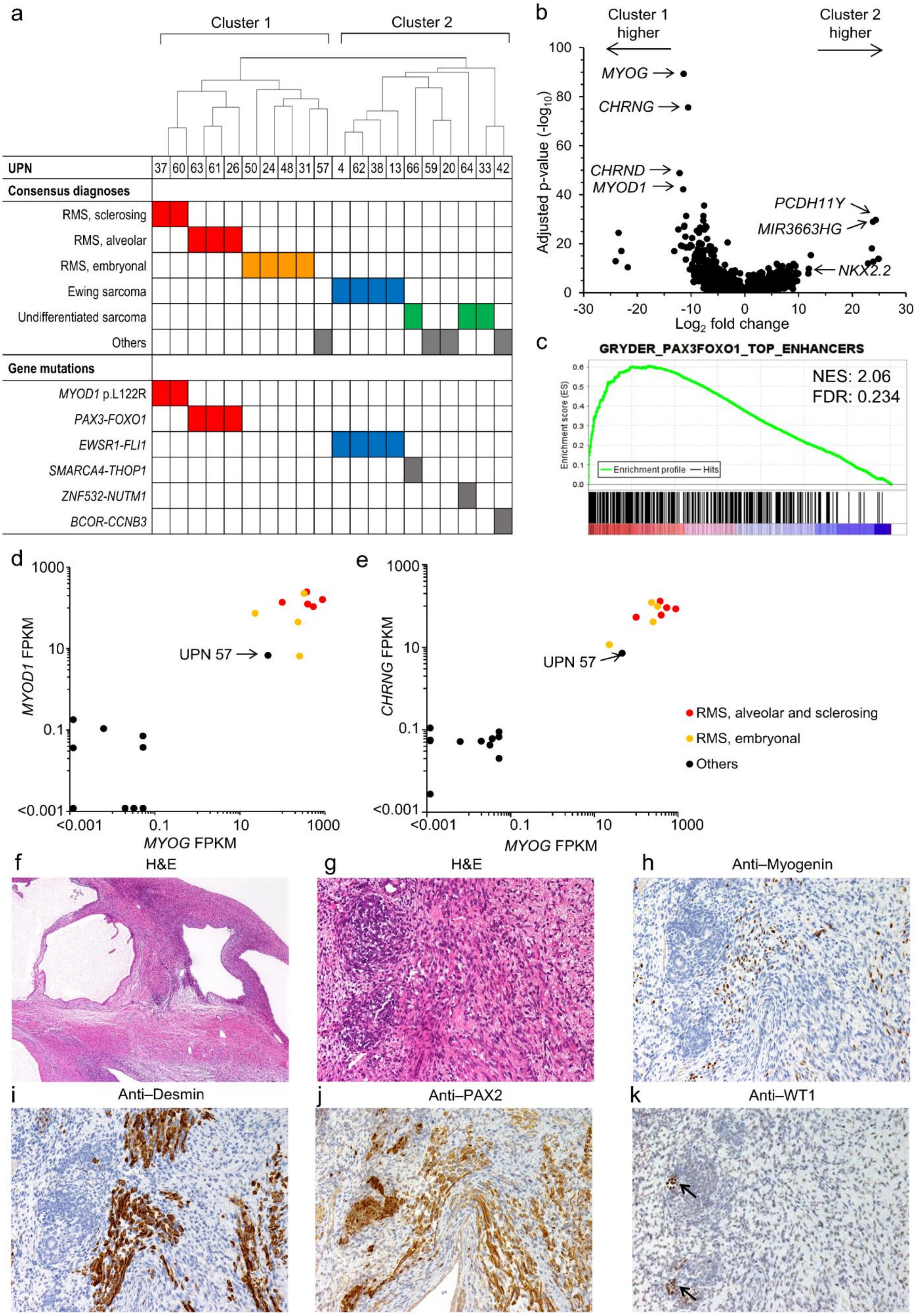

a
Cluster 1
Cluster 2
UPN
37 60 63 61 26 50 24 48 31 57 4 62 38 13 66 59 20 64 33 42
Consensus diagnoses
RMS, sclerosing
RMS, alveolar
RMS, embryonal
Ewing sarcoma
Undifferentiated sarcoma
Others
Gene mutations
MYOD1 p.L122R
PAX3-FOXO1
EWSR1-FLI1
SMARCA4-THOP1
ZNF532-NUTM1
BCOR-CCNB3
b
Cluster 1 higher
Cluster 2 higher
MYOG
CHRNG
CHRND
MYOD1
PCDH11Y
MIR3663HG
NKX2.2
Adjusted p-value (-log10)
Log2 fold change
c
GRYDER_PAX3FOXO1_TOP_ENHANCERS
NES: 2.06
FDR: 0.234
Enrichment score (ES)
Enrichment profile
Hits
d
MYOD1 FPKM
MYOG FPKM
UPN 57
e
CHRNG FPKM
MYOG FPKM
UPN 57
RMS, alveolar and sclerosing
RMS, embryonal
Others
f
H&E
g
H&E
h
Anti–Myogenin
i
Anti–Desmin
j
Anti–PAX2
k
Anti–WT1

Supplementary Figure 5. Gene expression-based clustering. (**a**) Using gene expression profiling, 20 patients were classified into two clusters: Cluster 1 (rhabdomyosarcoma group, n = 10) and Cluster 2 (Ewing sarcoma and others group, n = 10). (**b**) Volcano plot, comparing the expression profiles between Cluster 1 and Cluster 2. (**c**) Gene-set enrichment analysis (GSEA) revealed the marked enrichment of PAX3FOXO1_TOP_ENHANCERS-associated genes in Cluster 1. (**d** and **e**) Patients with rhabdomyosarcoma (n = 9) and UPN 57 showed higher *MYOG*, *MYOD1*, and *CHRNG* gene expression. (**f–k**) Histopathological features of UPN 57. The section shows blastemal cells, epithelial cells, and mesenchymal rhabdomyomatous cells. Immunostaining for myogenin, desmin, and PAX2 was positive, whereas WT1 staining was negative (black arrows shows lymphocytes).
UPN, unique patient number; RMS, rhabdomyosarcoma; NES, normalized enrichment score; FDR, false discovery rate; FPKM, fragments per kilobase of exon per million reads mapped.

Supplementary Table 1. Details of patient diagnoses

UPN	Age (Month)	Sex	Institutional diagnosis	Consensus diagnosis without RNA-seq information	Detected mutations	Consensus diagnosis with RNA-seq information
1	4	F	RMS, alveolar	RMS, alveolar	*PAX3-FOXO1*	RMS, alveolar
2	11	F	RMS, embryonal	RMS, embryonal		RMS, embryonal
3	122	F	RMS, embryonal	RMS, embryonal		RMS, embryonal
4	24	F	RMS, embryonal	ES	*EWS-FLI1*	ES
5	78	M	RMS, NOS	RMS, NOS		RMS, NOS
6	5	F	RMS, alveolar	RMS, alveolar		RMS, alveolar
7	3	M	RMS, NOS	RMS, spindle cell	*SRF-NCOA1*	RMS, spindle cell
8	141	M	RMS, alveolar	Undifferentiated sarcoma	*EWS-ATF1*	Clear cell sarcoma
9	45	M	ES	ES		ES
10	70	M	ES	ES	*EWS-FLI1*	ES
11	52	M	Langerhans cell histiocytosis	Langerhans cell histiocytosis		Langerhans cell histiocytosis
13	141	M	ES	ES	*EWS-FLI1*	ES
14	97	M	Myxopapillary ependymoma	Myxopapillary ependymoma		Myxopapillary ependymoma
15	48	F	Inflammatory pseudotumor	Inflammatory myofibroblastic tumor	*TPM4-ALK*	Inflammatory myofibroblastic tumor
16	90	M	MPNST	MPNST		MPNST
18	72	F	Myxopapillary ependymoma	Myxopapillary ependymoma		Myxopapillary ependymoma
19	185	F	SPTCL	SPTCL		SPTCL
20	10	M	Neuroblastoma	Neuroblastoma		Neuroblastoma
21	32	F	RMS, embryonal	RMS, alveolar	*PAX3-FOXO1*	RMS, alveolar
23	16	F	RMS, NOS	RMS, NOS		RMS, NOS
24	24	F	RMS, embryonal	RMS, embryonal		RMS, embryonal
25	32	M	RMS, embryonal	ES	*EWS-ETV1*	ES
26	18	F	RMS, embryonal	RMS, alveolar	*PAX3-FOXO1*	RMS, alveolar
27	87	M	RMS, embryonal	RMS, embryonal		RMS, embryonal
29	24	F	Malignant rhabdoid tumor	Malignant rhabdoid tumor		Malignant rhabdoid tumor
30	1	F	Malignant rhabdoid tumor	Malignant rhabdoid tumor		Malignant rhabdoid tumor
31	102	M	RMS, embryonal	RMS, embryonal		RMS, embryonal
33	15	F	Undifferentiated sarcoma	Undifferentiated sarcoma		Undifferentiated sarcoma

34	187	F	ES	ES	*EWS-FLI1*	ES
36	160	M	Extraskeletal myxoid chondrosarcoma	Undifferentiated sarcoma	*PTCH1-GLI1*	*GLI1*-rearranged tumor
37	89	M	RMS, alveolar	RMS, sclerosing	*MYOD1* p.L122R	RMS, sclerosing
38	215	F	ES	ES	*EWS-FLI1*	ES
42	177	M	Clear cell sarcoma of the kidney	Clear cell sarcoma of the kidney	*BCOR-CCNB3*	Clear cell sarcoma of the kidney (Sarcoma with *BCOR* genetic alterations)
46	199	F	Liposarcoma	Liposarcoma		Liposarcoma
48	25	M	RMS, embryonal	RMS, embryonal		RMS, embryonal
50	69	F	RMS, embryonal	RMS, embryonal		RMS, embryonal
51	3	M	RMS, embryonal	RMS, NOS		RMS, mixed phenotype
53	175	M	Pleuropulmonary blastoma	Undifferentiated sarcoma	*SS18-SSX2*	Synovial sarcoma
54	175	F	Synovial sarcoma	Synovial sarcoma	*SS18-SSX2*	Synovial sarcoma
57	6	F	Cystic partially differentiated nephroblastoma	Fetal rhabdomyomatous nephroblastoma		Fetal rhabdomyomatous nephroblastoma
59	55	M	Malignant rhabdoid tumor	Malignant rhabdoid tumor		Malignant rhabdoid tumor
60	92	F	RMS, alveolar	RMS, sclerosing	*MYOD1* p.L122R	RMS, sclerosing
61	136	M	RMS, embryonal	RMS, alveolar	*PAX3-FOXO1*	RMS, alveolar
62	125	M	ES	ES	*EWS-FLI1*	ES
63	179	F	RMS, alveolar	RMS, alveolar	*PAX3-FOXO1*	RMS, alveolar
64	23	M	Undifferentiated sarcoma	NUT carcinoma	*ZNF532-NUTM1*	NUT carcinoma
66	186	F	ALCL	Undifferentiated sarcoma	*SMARCA4-THOP1**	SMARCA4-deficient undifferentiated sarcoma with *SMARCA4-THOP1*

Abbreviations; RMS, rhabdomyosarcoma; ES, Ewing sarcoma; MPNST, malignant peripheral nerve sheath tumor; SPTCL, subcutaneous panniculitis-like T-cell lymphoma; NOS, not otherwise specified; ALCL, anaplastic large cell lymphoma

* novel fusion genes

Supplementary Table 2. List of genes for point mutation analysis

Disease	Genes	Mutations
Angiolipoma	*PRKD2*	
Fibrous hamartoma of infancy	*EGFR*	
Desmoid fibromatosis, Neuromuscular choristoma	*CTNNB1*	p.Thr41Ala<br>p.Ser45Pro<br>p.Ser45Phe
Anastomosing haemangioma	*GNAQ*<br>*GNA14*	
RMS, spindle cell / sclerosing	*MYOD1*	p.Leu122Arg
Granular cell tumour	*ATP6AP1*<br>*ATP6AP2*	
Intramuscular myxoma	*GNAS*	Exon 8 and 9
Extrarenal rhabdoid tumour	*SMARCB1*	
Chondroblastoma	*H3.3*	p.Lys36Met
Giant cell tumor of bone	*H3.3*	p.Gly34Trp
Fibrous dysplasia	*GNAS*	p.Arg201His<br>p.Arg201Cys
SMARCA4-DTS	*SMARCA4*	

Abbreviations; RMS, rhabdomyosarcoma; SMARCA4-DTS, SMARCA4-deficient thoracic sarcoma

Supplementary Table 3. Institutional diagnosis of entire cohort and analyzed cohort

Institutional diagnosis	Entire cohort n = 88	Analyzed patients n = 47
RMS	38 (43.2%)	22 (46.8%)
ES	13 (14.8%)	6 (12.8%)
Spinal cord tumor	7 (8.0%)	3 (6.4%)
Malignant rhabdoid tumor	4 (4.5%)	3 (6.4%)
CCSK	3 (3.4%)	1 (2.1%)
Synovial sarcoma	2 (2.3%)	1 (2.1%)
Undifferentiated sarcoma	2 (2.3%)	2 (4.3%)
Undifferentiated sarcoma of the liver	2 (2.3%)	-
ALCL	1 (1.1%)	1 (2.1%)
Ameloblastoma	1 (1.1%)	-
Cystic partially differentiated nephroblastoma	1 (1.1%)	1 (2.1%)
DSRCT	1 (1.1%)	-
Extraskeletal myxoid chondrosarcoma	1 (1.1%)	1 (2.1%)
Ganglioglioma	1 (1.1%)	-
Inflammatory pseudotumour	1 (1.1%)	1 (2.1%)
LCH	1 (1.1%)	1 (2.1%)
Liposarcoma	1 (1.1%)	1 (2.1%)
Neuroblastoma	1 (1.1%)	1 (2.1%)
Pleuropulmonary blastoma	1 (1.1%)	1 (2.1%)
SPTCL	1 (1.1%)	1 (2.1%)
Others	5 (5.7%)	-

Abbreviations; RMS, rhabdomyosarcoma; ES, Ewing sarcoma; CCSK, Clear cell sarcoma of the kidney; ALCL, Anaplastic large cell lymphoma; DSRCT, Desmoplastic small round cell tumor; LCH, Langerhans cell histiocytosis; SPTCL, subcutaneous panniculitis-like T-cell lymphoma

Supplementary Table 4. Details of discrepancy between institutional diagnosis and consensus diagnosis without RNA-seq information

UPN	Institutional diagnosis	Consensus diagnosis without RNA-seq information	Reasons for diagnostic discrepancies
4	RMS, embryonal	ES	Improvements in pathological diagnostic techniques
7	RMS, NOS	RMS, spindle cell	Changes in pathological classification over time
8	RMS, alveolar	Undifferentiated sarcoma	Improvements in pathological diagnostic techniques
15	Inflammatory pseudotumor	Inflammatory myofibroblastic tumor	Changes in pathological classification over time
21	RMS, embryonal	RMS, alveolar	Improvements in pathological diagnostic techniques
25	RMS, embryonal	ES	Improvements in pathological diagnostic techniques
26	RMS, embryonal	RMS, alveolar	Improvements in pathological diagnostic techniques
36	Extraskeletal myxoid chondrosarcoma	Undifferentiated sarcoma	Improvements in pathological diagnostic techniques
37	RMS, alveolar	RMS, sclerosing	Changes in pathological classification over time
51	RMS, embryonal	RMS, NOS	Improvements in pathological diagnostic techniques
53	Pleuropulmonary blastoma	Undifferentiated sarcoma	Improvements in pathological diagnostic techniques
57	Cystic partially differentiated nephroblastoma	Fetal rhabdomyomatous nephroblastoma	Improvements in pathological diagnostic techniques
60	RMS, alveolar	RMS, sclerosing	Changes in pathological classification over time
61	RMS, embryonal	RMS, alveolar	Improvements in pathological diagnostic techniques
64	Undifferentiated sarcoma	NUT carcinoma	Improvements in pathological diagnostic techniques
66	ALCL	Undifferentiated sarcoma	Improvements in pathological diagnostic techniques

Abbreviations; RMS, rhabdomyosarcoma; ES, Ewing sarcoma; ALCL, Anaplastic large cell lymphoma; NOS, not otherwise specified;

Supplementary Table 5. List of primers for RT-PCR

Disease	Target gene alteration	Forward primer (5' - 3')	Reverse primer (5' - 3')
Ewing sarcoma	*EWSR1-FLI1*	GAAGAGGGGGATTTGATCGT	GGACTTTTGTTGAGGCCAGA
	EWSR1-ETV1	ACAGCCAAGCTCCAAGTC	TGTGGGTCCTTCCCGATAC
Rhabdomyosarcoma	*PAX3-FOXO1*	CCGACAGCAGCTCTGCCTAC	TGAACTTGCTGTGTAGGGACAG
	SRF-NCOA1	CAGTGCAGGCCATTCAAGTG	GTGGGTGGACAGAGAAGCTC
	MYOD1 p.L122R	CAAGCGCAAGACCACCAAC	GGTTTGGATTGCTCGACGTG
Clear cell sarcoma	*EWSR1-ATF1*	AGAGCGAGGTGGCTTCAATA	CAACTGTAAGGCTCCATTTGGG
Clear cell sarcoma of the kidney	*BCOR-CCNB3*	GGCTCCACCCCAGTGATCT	GGGTGTTTTGGAGGTGGTGGAT
GLI1-rearranged tumor	*PTCH1-GLI1*	GAAATCCAAGCCCAGCGT	AGGAAATGCGATCTGTGATG
Inflammatory myofibroblastic tumor	*TPM4-ALK*	TGACCGCAAATACGAGGAGG	GAGGGTGATGTTTTTCCGCG
NUT carcinoma	*ZNF532-NUTM1*	GGAAACCAAGAAAGTGGCCAG	TCCATGAACCTTTCTGCCATCT
SMARCA4 deficient undifferentiated	*SMARCA4-THOP1*	CATTGAGCTCAAGGCCCTCA	CGTCCTCCCTCATGCTCATC
sarcoma	*SMARCA4* c.3168+1	ACTCTTCCCCACTAGAGCGT	AAGCACTGGTGAAGCGAAGA
Synovial sarcoma	*SS18-SSX2*	AGGTCAGCAGTATGGAGGATATAGACC	CACTTCCTCCGAATCATTTCCT

Supplementary References

1 Oue, T. *et al.* Outcome of pediatric renal tumor treated using the Japan Wilms Tumor Study-1 (JWiTS-1) protocol: a report from the JWiTS group. *Pediatr Surg Int* **25**, 923-929, doi:10.1007/s00383-009-2449-0 (2009).

2 Pierron, G. *et al.* A new subtype of bone sarcoma defined by BCOR-CCNB3 gene fusion. *Nat Genet* **44**, 461-466, doi:10.1038/ng.1107 (2012).
